# RETRACTION: Clinical Analysis of 10 cases With Subcutaneous Panniculitis‐like T‐cell Lymphoma and Tissue AURKA Expression

**DOI:** 10.1111/srt.70230

**Published:** 2025-09-18

**RETRACTION**: Z. Zheng, J. Teng, M. Zeng, and C. Lu, “Clinical Analysis of 10 cases With Subcutaneous Panniculitis‐like T‐cell Lymphoma and Tissue AURKA Expression,” *Skin Res Technol*. 2024;30(8):e13899. doi:10.1111/srt.13899.

The above article, published online on 7 August 2024 in Wiley Online Library (wileyonlinelibrary.com), has been retracted by agreement between *Skin Research and Technology*; and John Wiley & Sons Ltd. The article was accepted for publication following an insufficient peer review process that does not meet the standards of the journal's peer review policy. Furthermore, the study's rationale lacks support from the literature, and the conclusions are not supported by the data. Accordingly, the article has been retracted. The authors disagree with the decision of retraction.

